# A Line-Source Approach for Simulating MammoWave Microwave Imaging Apparatus for Breast Lesion Detection

**DOI:** 10.3390/s25123640

**Published:** 2025-06-10

**Authors:** Navid Ghavami, Sandra Dudley, Mohammad Ghavami, Gianluigi Tiberi

**Affiliations:** 1 UBT—Umbria Bioengineering Technologies, 06081 Perugia, Italy; n.ghavami@ubt-tech.com; 2 School of Engineering, London South Bank University, London SE1 0AA, UK; dudleyms@lsbu.ac.uk (S.D.); ghavamim@lsbu.ac.uk (M.G.)

**Keywords:** biomedical electromagnetic imaging, electromagnetic analysis, electromagnetic propagation, microwave imaging

## Abstract

Here, we propose an analytical approach to simulating MammoWave, a novel apparatus for breast cancer detection using microwave imaging. The approach is built upon the theory of cylindrical waves emitted by line sources. The sample is modelled as a cylinder with an inclusion. Our results indicate that when compared with phantom measurements, our approach gives an average relative error (between the image generated through measurement with phantoms and the image generated through the analytical simulation approach) of less than 6% when considering the full frequency band of 1–9 GHz. The procedure permits the simulation of the MammoWave imaging system loaded with multilayered eccentric cylinders; thus, it can be used to obtain an insight into MammoWave’s detection capability, without having to perform either time-consuming full-wave simulations or phantom measurements.

## 1. Introduction

Microwave imaging has garnered increasing interest for biomedical applications, particularly for its potential in breast cancer detection. This interest is driven by the substantial difference in dielectric properties between malignant and normal tissues within the microwave frequency band (1–10 GHz) [1].

Mammography is the gold-standard technology for breast cancer screening. However, it has some limitations and potential harms, such as the use of ionizing radiation, breast compression, and performance restrictions due to the intrinsic nature of X-rays. Considering other established techniques, magnetic resonance imaging (MRI) entails long scan times, while ultrasound encounters challenges in imaging objects with substantial impedance differences [2].

Ongoing research in microwave breast imaging is primarily categorized into two main approaches: tomographic-based and radar-based techniques. Globally, there are several research groups investigating the use of microwave imaging for breast cancer detection [3,4]. Among these, only a few groups have reached the advanced clinical trial stage, including MammoWave developed by UBT (Italy) [5], which has already been used in three prospective clinical trials (Clinicaltrials.gov identifiers NCT04253366, NCT05300464, and NCT06291896, the latest is still ongoing) on thousands of women [6,7]. Other prototypes reaching the clinical validation stage include Dartmouth University (USA), Micrima (University of Bristol, UK), Wavelia by MVG (France), and SAFE by Mitos Medical Technologies (Turkey). Since the 1990s, Dartmouth has tested its prototype on 500 subjects in the USA [8]. However, their research (based on a tomographic approach) was abandoned after a few years. Micrima developed MARIA™, which has been tested on >500 subjects, only symptomatic [9]. MammoWave presents key technological differences compared to MARIA: MARIA employs an ultra-wide band (UWB) radar technique. UBT’s system uses Huygens’ principle. A comparison between the two approaches has been conducted, showing that UBT’s reconstruction method results in improvements in the localization of the tumor (offset lower than 1 mm) and in signal-to-clutter ratio (up to 0.6 dB), as well as in more robustness to sparse data [10]. Moreover, MARIA needs a matching liquid between the breast and the antennas to ensure the system’s dielectric continuity. MammoWave works with a system with the antennas in air, providing a simpler and more comfortable experience for the patient. Finally, MammoWave uses only two antennas compared to MARIA’s 60, reducing manufacturing and maintenance costs. Wavelia completed its pilot clinical trials on ∼100 subjects [11]. Its system uses matching liquid to perform examination. Mitos has also recently started its feasibility clinical trials; up to now, 113 patients have undergone the scanning procedure using SAFE [12].

Before and in adjunct to clinical validation, microwave imaging devices require detailed investigation, optimization, and performance quantification; such activities include both the hardware [13], i.e., the components of the radio-frequency transmission and reception chain, and imaging algorithms [3]. Electromagnetic modeling and simulation are often used prior to or in combination with phantom measurements.

The electromagnetic interactions between microwave imaging antennas and objects (to be imaged) cannot typically be solved through analytical approaches. Consequently, full-wave simulators based on Maxwell’s equation solutions are implemented and widely used [14,15]. However, tailored analytical approaches may be created to calculate such interactions by employing simplified assumptions and geometries. Some examples can be found in other medical imaging applications [16,17].

Here, we propose the simulation of MammoWave via an analytical approach built upon the theory of cylindrical waves radiated from a line source [18]. The sample will be modelled as a cylinder with an inclusion [19]. It is worthwhile pointing out that the (frequency-varying) dielectric properties of both the cylinder and the inclusion will be appropriately set to mimic the dielectric properties of different conditions of breast tissues, according to the literature [20].

The novelty of the proposed work lies in the fact that this analytical procedure can enable us to have an insight into MammoWave’s detection capability without having to perform either time-consuming full-wave simulation or phantom experiments. In Section 2, we describe the MammoWave device and the proposed analytical procedure. Section 3 presents the simulation and measurements results, and finally, Section 4 concludes the paper.

## 2. Materials and Methods

### 2.1. MammoWave Description

**Figure 1:**
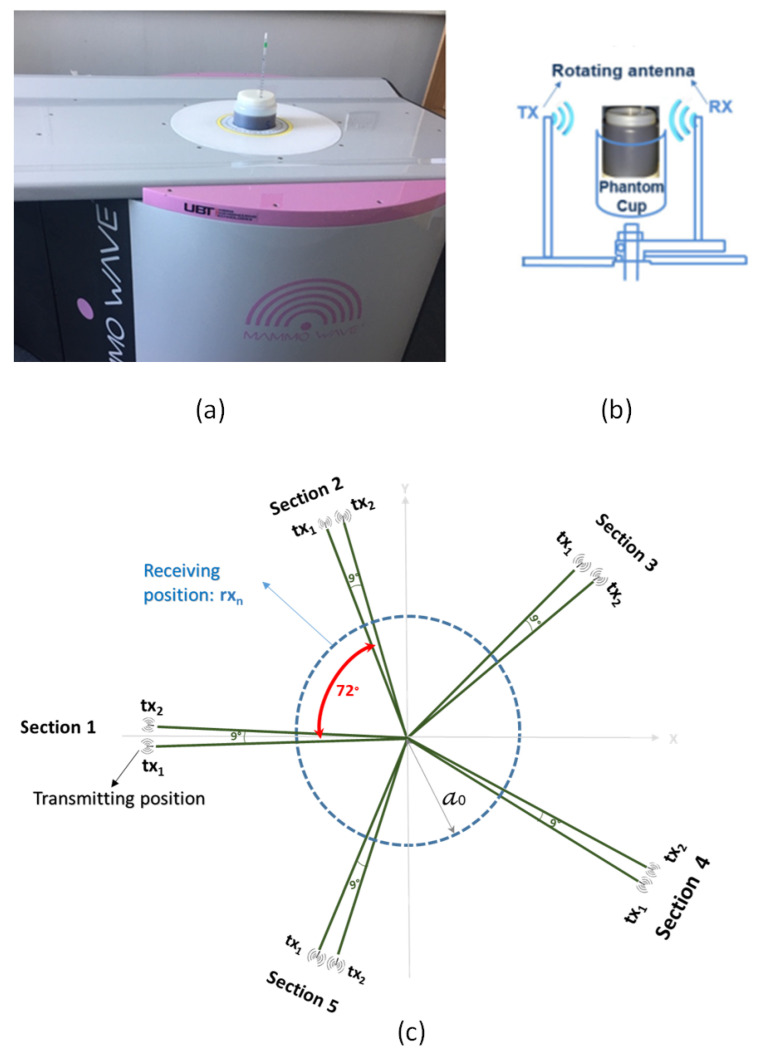
(**a**) MammoWave device (with the phantom), (**b**) measurement setup showing the placement of antennas and the phantom inside MammoWave, and (**c**) MammoWave’s antenna configuration.

The MammoWave device (Figure 1) is constituted by an aluminum cylindrical hub. The device’s internal surfaces are shielded with absorbing material. The instrument (appropriately integrated with a bed) is additionally constituted by a hub with a cup that is placed to contain the breast of the patient (prone position) or, in this case, the phantoms, and two arms to associate the transmitter (tx) and the receiver (rx) to the hub through rotation.

The transmitting antenna is a Horn-type with the voltage standing wave ratio (VSWR) < 3 in the frequency band of 1–9 GHz, and the receiving antenna is a Vivaldi-type with VSWR < 3 in the same frequency band. The Tx and rx antennas are connected to a 2-port Cobalt C1209 vector network analyzer (VNA), operating up to 9 GHz.

Both antennas are mounted at the same height, rotating around the azimuth, allowing them to measure S21 from distinct angular locations. Thus, S21 measurements can be performed through a multi-bistatic mode, recording S21 for each rx and tx position. In more detail, for each transmitting position, the receiver moves on a circumference with a radius of $a_{0}$ = 7 cm to measure the received signals in the $N_{PT}=80$ position equally displaced along the azimuth, i.e., with an angular step of 4.5${\,}^{\circ }$. We can denote the rx position as ${\mathrm{rx}}_{np}\equiv \left(a_{0},\phi_{np}\right)\equiv {\vec{\rho }}_{np}$, with n = 1, 2, …, 80. Moreover, regarding tx, the experiments presented here have been performed by using 10 transmitting positions, divided into five groups centered at 0, 72, 144, 216, and 288 degrees respectively; each group, i.e., section, contains two transmitting positions 9${\,}^{\circ }$ apart.

Using the VNA, we record complex S21 signals (1–9 GHz, step of 5 MHz) for each position of tx and rx. This S21 signal can be expressed as $S21_{n}^{m,p}\left(a_{0},\phi_{n};{\mathrm{tx}}_{m,p};f\right)$, where *m* = 1, 2, …, 5 specifies tx sections. Moreover, *f* stands for frequency, and *p* = 1,2 and p’ = 1,2 denote the position within each transmitting doublet.

An algorithm based on Huygens’ principle (HP) is then utilized for processing the received signals in order to calculate the object’s internal field; this internal field is subsequently used to produce an image representing the homogeneity level of dielectric properties. As a final step, to reduce the artefacts, we subtract S21 signals obtained from 2 measurements acquired from doublets belonging to the same section [5]:

(1) $$\begin{array}{cc} E_{\mathrm{HP},2D}^{\mathrm{rcstr}}\left(\rho ,\phi ;{\mathrm{tx}}_{m,p}-{\mathrm{tx}}_{m,p^{\prime }};f\right)\propto & \sum_{n=1}^{N_{PT}}\left(S21_{n}^{m,p}\left(a_{0},\phi_{n};{\mathrm{tx}}_{m,p};f\right)-S21_{n}^{m,p^{\prime }}\left(a_{0},\phi_{n};{\mathrm{tx}}_{m,p^{\prime }};f\right)\right)\\ & G(k_{1}|\vec{\rho_{n}}-\vec{\rho }|) \end{array}$$

In the above, $\left(\rho ,\phi \right)\equiv \vec{\rho }$ denotes the observation spot, and Δs specifies spatial sampling. Additionally, *G* symbolizes Green’s function, and $k_{1}$ is the wavenumber. The strings rcstr and HP are short for the reconstructed internal field and Huygens’ principle procedure, respectively. The permittivity value of wavenumber $k_{1}$ is set to that of free space due to the existence of both antennas in air [4].

Assuming we consider $N_{F}$ frequencies $f_{i}$ within frequency band *B*, the final intensity image (Img) can be achieved using Equation (2), via incoherent addition of all solutions of all 5 sections:

(2) $$\mathrm{Img}\left(\rho ,\phi \right)=\sum_{m=1}^{5}\sum_{\begin{array}{c} p=1\\ p^{\prime }=1\\ p\neq p^{\prime } \end{array}}^{2}\sum_{i=1}^{N_{F}}{\left|E_{\mathrm{HP},2D}^{\mathrm{rcstr}}\left(\rho ,\phi ;{\mathrm{tx}}_{m,p}-{\mathrm{tx}}_{m,p^{\prime }};f_{i}\right)\right|}^{2}$$

The images obtained through Equation (2) are two-dimensional (2D) images in the azimuthal (coronal) plane. Images are in arbitrary units and are normalized to average.

MammoWave’s ability to detect inclusions has been demonstrated via measurements on phantoms, which may be constituted by a plastic cylinder (used as a container) with inclusions, i.e., cylindrical tubes filled with appropriate liquids [5].

### 2.2. Line-Source Theory

Let us focus on an electric current line source (I) situated at $\left(\rho^{\prime },\phi^{\prime }\right)$ in free space. The electric current line is parallel to the z-axis. The field can be obtained by allowing $\vec{A}=\psi {\hat{i}}_{z}$ and using the following relations [18]:

(3) $$\begin{array}{c} \vec{E}\left(\vec{\rho }\right)=\left[\begin{array}{c} \frac{1}{j\omega \varepsilon_{0}}\frac{\delta^{2}\psi }{\delta \rho \delta z}{\hat{i}}_{\rho }\\ \frac{1}{j\omega \varepsilon_{0}}\frac{1}{\rho }\frac{\delta^{2}\psi }{\delta \phi \delta z}{\hat{i}}_{\phi }\\ \frac{1}{j\omega \varepsilon_{0}}\left(\frac{\delta^{2}}{\delta z^{2}}+k_{0}^{2}\right)\psi {\hat{i}}_{z} \end{array}\right];\;\vec{H}\left(\vec{\rho }\right)=\left[\begin{array}{c} \frac{1}{\rho }\frac{\delta \psi }{\delta \phi }{\hat{i}}_{\rho }\\ -\frac{\delta \psi }{\delta \rho }{\hat{i}}_{\phi }\\ 0{\hat{i}}_{z} \end{array}\right] \end{array}$$

where $k_{0}$ is the free space wavenumber, and ω=2πf, where *f* is the operation frequency. It holds that

(4) $$\vec{A}=\frac{I}{4j}H_{0}^{\left(2\right)}(k_{0}|\vec{\rho }-{\vec{\rho }}^{\prime }\left|\right){\hat{i}}_{z}$$

Making use of the addition theorem, we obtain

(5) $$\begin{array}{c} H_{0}^{\left(2\right)}(k_{0}|\vec{\rho }-{\vec{\rho }}^{\prime }\left|\right)=\{\begin{array}{c} \sum_{n=-\infty }^{+\infty }H_{n}^{\left(2\right)}\left(k_{0}\rho^{\prime }\right)J_{n}\left(k_{0}\rho \right)e^{jn(\phi -\phi^{\prime })}\;\mathrm{for}\;\rho <\rho^{\prime }\\ \sum_{n=-\infty }^{+\infty }H_{n}^{\left(2\right)}\left(k_{0}\rho \right)J_{n}\left(k_{0}\rho^{\prime }\right)e^{jn(\phi -\phi^{\prime })}\;\mathrm{for}\;\rho >\rho^{\prime } \end{array} \end{array}$$

**Figure 2:**
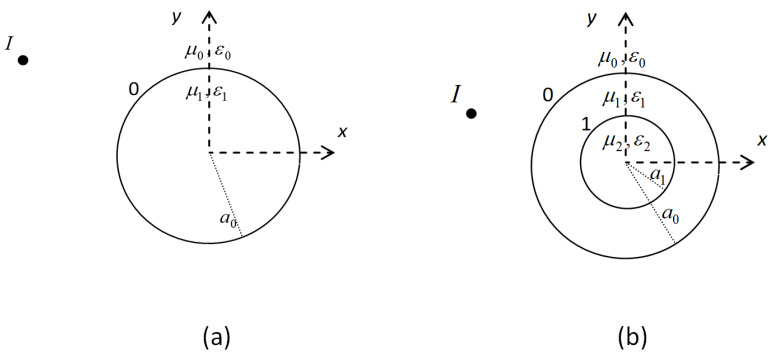
Homogeneous (**a**) and stratified (2-layer) concentric dielectric cylinder (**b**) in free space imaged using a line source *I*.

Let us consider now a 2-layer stratified dielectric cylinder with external radius $a_{1}$ illuminated by the line source radiating in free space (Figure 2b) [18]. The cylinder is positioned at the center of the reference system. The external layer is characterized by relative constants $\varepsilon_{r1},\mu_{r1}$, and by conductivity $\sigma_{1}$. It holds ($\mu_{r1}=1$) that

(6) $$\begin{array}{c} \left\{ \begin{array}{c} \varepsilon_{1}=\varepsilon_{0}\varepsilon_{eq1}\\ \mu_{1}=\mu_{0} \end{array},\;\mathrm{with}\;\varepsilon_{eq1}=\left(\varepsilon_{r1}-j\frac{\sigma_{1}}{2\pi f\varepsilon_{0}}\right)\right. \end{array}$$

where $k_{1}$ is the internal wavenumber of the cylinder, defined as

(7) $$k_{1}=\sqrt{{\left(2\pi f\right)}^{2}\varepsilon_{1}\mu_{1}}$$

We can characterize this internal layer through relative constants $\varepsilon_{r2},\mu_{r2}$, and by conductivity $\sigma_{2}$. It holds ($\mu_{r2}=1$) that

(8) $$\begin{array}{c} \left\{ \begin{array}{c} \varepsilon_{2}=\varepsilon_{0}\varepsilon_{eq2}\\ \mu_{2}=\mu_{0} \end{array},\;\mathrm{with}\;\varepsilon_{eq2}=\left(\varepsilon_{r2}-j\frac{\sigma_{2}}{2\pi f\varepsilon_{0}}\right)\right. \end{array}$$

(9) $$k_{2}=\sqrt{{\left(2\pi f\right)}^{2}\varepsilon_{2}\mu_{2}}$$

The frequency dependence of $\varepsilon_{r1}$ and $\sigma_{1}$ may be taken into account as well.

We proceed by writing the following equations:

(10) $$\begin{array}{cc} \; & E_{z}^{0}\propto \sum_{n=-\infty }^{+\infty }H_{n}^{\left(2\right)}\left(k_{0}\rho^{\prime }\right)J_{n}\left(k_{0}\rho \right)e^{jn(\phi -\phi^{\prime })}+\sum_{n=-\infty }^{+\infty }a_{n}H_{n}^{\left(2\right)}\left(k_{0}\rho \right)e^{jn(\phi -\phi^{\prime })}\\ \; & E_{z}^{1}\propto \sum_{n=-\infty }^{+\infty }b_{n}J_{n}\left(k_{1}\rho \right)e^{jn\phi }+\sum_{n=-\infty }^{+\infty }c_{n}H_{n}^{\left(2\right)}\left(k_{1}\rho \right)e^{jn\phi }\\ \; & E_{z}^{2}\propto \sum_{n=-\infty }^{+\infty }d_{n}J_{n}\left(k_{2}\rho \right)e^{jn\phi } \end{array}$$

with other components related to $\vec{E}$ being 0, as derived from Equation (3). $k_{1}$ and $k_{2}$ denote wavenumbers inside the two cylindrical layers. The equations in (10) represent the free space inside cylinder #0 and inside cylinder #1, respectively. Similarly, Equations (11) and (12) hold for *H* as follows:

(11) $$\begin{array}{cc} \; & H_{\rho }^{0}\propto \frac{e^{jn\phi }}{\rho }\sum_{n=-\infty }^{+\infty }H_{n}^{\left(2\right)}\left(k_{0}\rho^{\prime }\right)J_{n}\left(k_{0}\rho \right)e^{jn(\phi -\phi^{\prime })}+\frac{e^{jn\phi }}{\rho }\sum_{n=-\infty }^{+\infty }a_{n}H_{n}^{\left(2\right)}\left(k_{0}\rho \right)e^{jn(\phi -\phi^{\prime })}\\ \; & H_{\rho }^{1}\propto \frac{e^{jn\phi }}{\rho }\sum_{n=-\infty }^{+\infty }b_{n}J_{n}\left(k_{1}\rho \right)e^{jn\phi }+\frac{e^{jn\phi }}{\rho }\sum_{n=-\infty }^{+\infty }c_{n}H_{n}^{\left(2\right)}\left(k_{1}\rho \right)e^{jn\phi }\\ \; & H_{\rho }^{2}\propto \frac{e^{jn\phi }}{\rho }\sum_{n=-\infty }^{+\infty }d_{n}J_{n}\left(k_{2}\rho \right)e^{jn\phi } \end{array}$$

and

(12) $$\begin{array}{cc} \; & H_{\phi }^{0}\propto -\sum_{n=-\infty }^{+\infty }H_{n}^{\left(2\right)}\left(k_{0}\rho^{\prime }\right)J_{n}^{\prime }\left(k_{0}\rho \right)e^{jn(\phi -\phi^{\prime })}+\sum_{n=-\infty }^{+\infty }a_{n}H_{n}^{{\,}^{\prime }\left(2\right)}\left(k_{0}\rho \right)e^{jn(\phi -\phi^{\prime })}\\ \; & H_{\phi }^{1}\propto -\sum_{n=-\infty }^{+\infty }b_{n}J_{n}^{\prime }\left(k_{1}\rho \right)e^{jn\phi }+{\frac{e^{jn\phi }}{\sum }}_{n=-\infty }^{+\infty }c_{n}H_{n}^{{\,}^{\prime }\left(2\right)}\left(k_{1}\rho \right)e^{jn\phi }\\ \; & H_{\phi }^{2}\propto -\sum_{n=-\infty }^{+\infty }d_{n}J_{n}^{\prime }\left(k_{2}\rho \right)e^{jn\phi } \end{array}$$

with other components related to $\vec{H}$ being 0, as derived through Equation (3).

All coefficients may be calculated through imposing boundary conditions of $\vec{H}$ and $\vec{E}$ on the two surfaces, and by using exponential functions’ orthogonality. In this way, the fields $\vec{H}$ and $\vec{E}$ could be calculated for each region. The aforementioned formulation was derived assuming a concentric 2-layer dielectric cylinder situated centrally within the reference system. Yet, this procedure could be easily extended to internal cylinders relocated arbitrarily respective to a reference system [21].

Equations (10)–(12) are denoted using a summation where *n* ranges from −∞ to +∞. For computational purposes, this summation is truncated after *N* terms (with *n* ranging from −*N* to *+N*). The value of *N* is decided such that convergence is reached; note that its value depends on both frequency and radius $a_{0}$. For the problems encountered here, we obtained *N* = 30.

### 2.3. Mammowave Simulation

Let us consider now the MammoWave microwave imaging device. We assume that the *M* different positions of the transmitting antennas can be represented by *M* electric current line sources. For each transmitting position, the signals S21 measured at $N_{PT}$ positions by the receiving antenna are proportional to $E_{z}^{0}$ calculated at ${\mathrm{rx}}_{np}\equiv \left(a_{0},\phi_{np}\right)\equiv {\vec{\rho }}_{np}$ and, thus, it can be simulated via Equation (10) if the geometrical and dielectric properties of the phantoms are known. Simulations are performed from 1 to 9 GHz, using a step of 5 MHz. More specifically, for each electric line source (representing a given position of the transmitting antenna) and for each frequency sample, the simulated $E_{z}^{0}$ is evaluated at ${\mathrm{rx}}_{np}\equiv \left(a_{0},\phi_{np}\right)\equiv {\vec{\rho }}_{np}$ (representing the positions of the receiving antenna). Next, the simulated $E_{z}^{0}$ is used in Equations (1) and (2) to generate a simulated image.

## 3. Results

**Figure 3:**
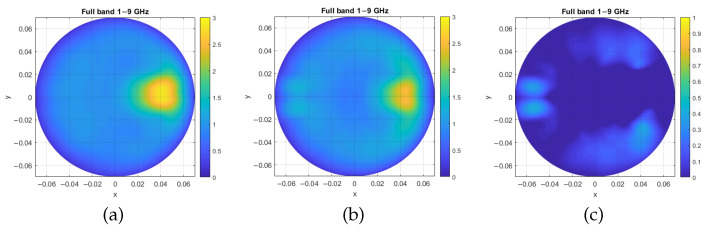
Test case 1, with inclusion placed at 0${\,}^{\circ }$: (**a**) image generated through measurement with phantom, (**b**) image generated through the analytical simulation approach, and (**c**) intensity map of the relative error between the analytical and measured images. Axis units are meters, while intensity maps are in arbitrary units.

For validation purposes, we performed MammoWave phantom measurements, using as a container a plastic cylinder (1 mm thick) with a height of 23 cm and diameter of 11 cm. The samples used for examination were sunflower oil with inclusions. To replicate the inclusions, 6 mm diameter tubes with 23 cm height were filled with 15% water and 85% glycol solution. The dielectric properties of oil and glycol water solution can be found in [5]; specifically, the dielectric properties of the oil used in this study may be considered a good approximation of the dielectric properties of high-adipose-content healthy breast tissue, while the dielectric properties of the glycol/water solution may be considered a good approximation of the dielectric properties of a breast lesion [5]. Measured S21 was then used in Equations (1) and (2) to generate an image, shown in Figure 3a.

Figure 3b refers to the image generated when using the simulated $E_{z}^{0}$ in Equations (1) and (2), obtained following the analytical simulation approach given in the previous section. Finally, in Figure 3c, a map of the relative error is given. In Figure 3, the full frequency band of 1–9 GHz is investigated.

**Figure 4:**
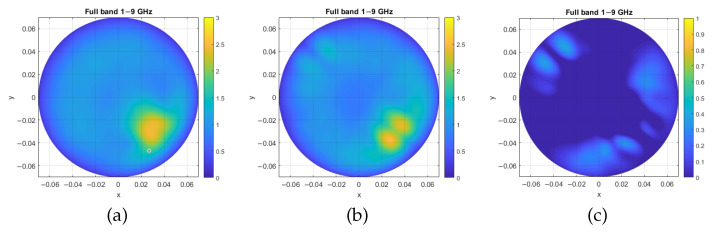
Test case 2, with inclusion placed at −45${\,}^{\circ }$: (**a**) image generated through measurement with phantom, (**b**) image generated through the analytical simulation approach, and (**c**) intensity map of the relative error between the analytical and measured images. Axis units are meters, while intensity maps are in arbitrary units.

To confirm that the detected peak is indeed the inclusion, we repeated the procedure, this time placing the inclusion for both the analytical simulation and the phantom measurement at −45${\,}^{\circ }$ relative to the x-axis. Figure 4a represents the images obtained through measurement, while Figure 4b,c display the image generated through the analytical simulation approach and the map of the relative error, respectively.

**Figure 5:**
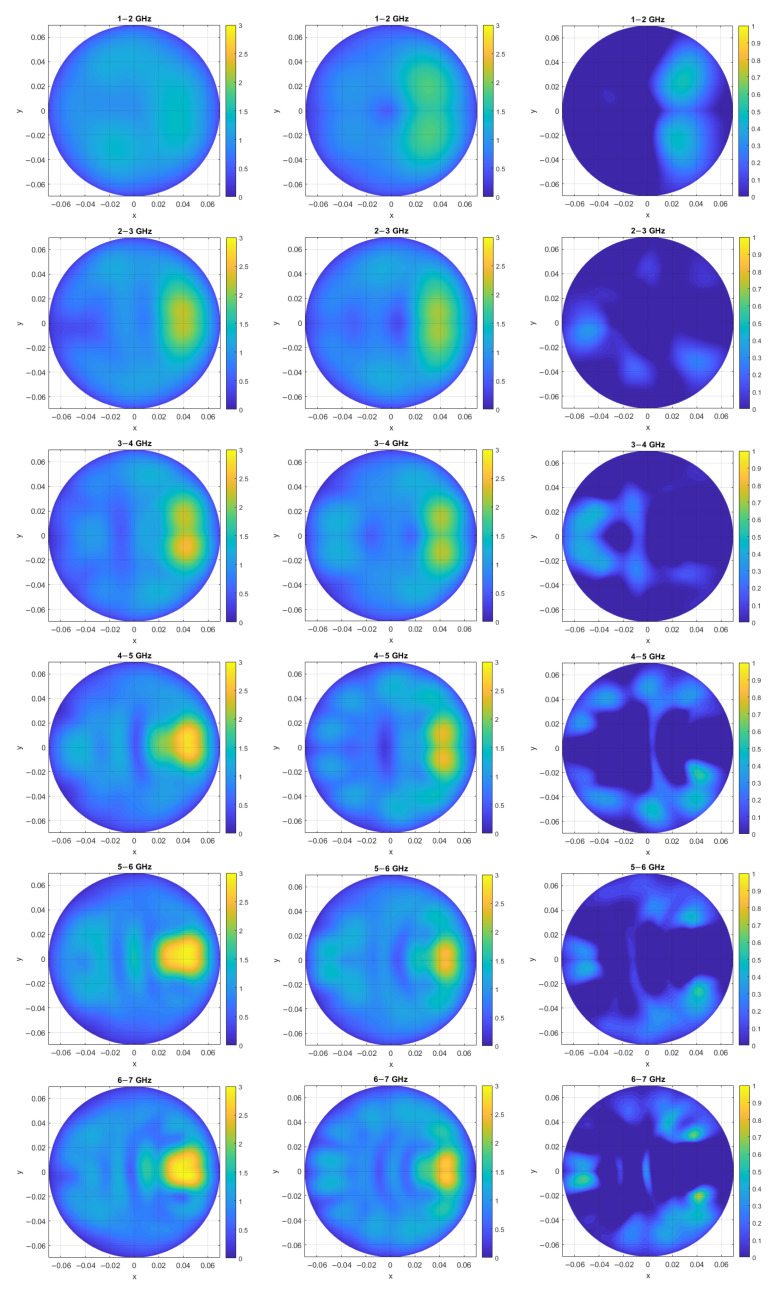

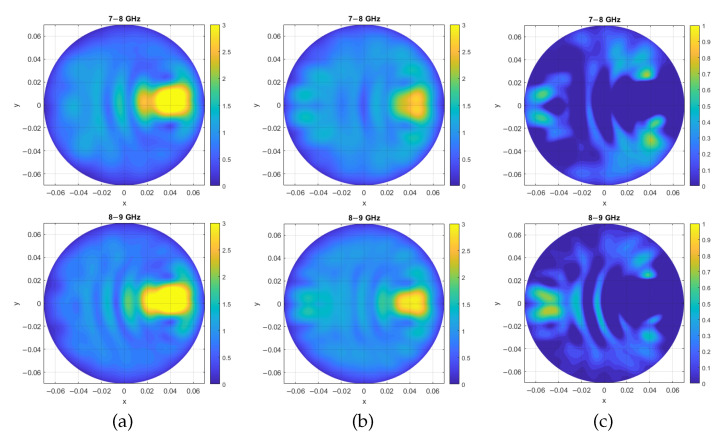
(**a**) Images generated through measurement with phantom for the eight 1 GHz sub-bands, (**b**) images generated through the analytical simulation approach for the eight 1 GHz sub-bands, and (**c**) intensity maps of the relative error between analytical and measured images for the eight 1 GHz sub-bands. Axis units are meters, while intensity maps are in arbitrary units.

Subsequently, we performed an additional investigation to observe the effect of individual 1 GHz sub-bands on the relative error. Figure 5a, from top to bottom, depicts the images obtained through measurement for individual sub-bands starting from 1–2 GHz and ending at 8–9 GHz, respectively. Similarly, the rows of Figure 5b display images generated through the analytical simulation approach for the same sub-bands. Finally, Figure 5c shows the relative error map corresponding to the eight individual sub-bands. It should be noted that, for each sub-band, the corresponding dielectric properties specific to that frequency range were used [22].

## 4. Discussions and Conclusions

Breast microwave imaging operates without ionizing radiation, using radio-frequency signals to discriminate between healthy and malignant breast tissues based on their dielectric properties. Moreover, breast microwave imaging devices do not require breast compression, making the examination more comfortable. Table 1 shows a general comparison between microwave imaging prototypes which have reached the advanced clinical trial stage [23].

In all the devices, the S21 parameter, which is the physical (complex) quantity measured by the VNA, contains the information about measured scattered fields. In MammoWave, this information is directly processed via HP to generate the images, which are intensity maps indicating the homogeneity level of dielectric properties. Intensity map images are expressed in arbitrary units; thus, they do not represent physical quantities. Images of measured S21 data can be obtained without any other a priori knowledge/processing, including antennas’ parameters and coupling; however, they do depend on antennas’ parameters and coupling since antennas’ parameters and coupling affect the S21. The simulator presented here allows for simulation of the scattered field, i.e., $E_{z}^{0}$, which may be then directly processed via HP to generate images. Simulation is performed using an analytical approach based on theory of cylindrical waves radiated by line currents. The approach permits the simulation of the MammoWave imaging system loaded with multilayered eccentric cylinders. The procedure has been verified through comparison with measurements, giving an average relative error of <6% between the image generated through measurement with the phantom and the image generated through the analytical simulation approach, when considering a full frequency band of 1–9 GHz.

The relative error between the maximum of the image (normalized to average) generated through measurement with the phantom and the maximum of the image (normalized to average) generated through the analytical simulation approach is <10%. It has been shown in [5] that the maximum of the image (normalized to average) is used to measure the non-homogenous behavior of images and can quantify MammoWave’s detection capability [5]. It follows that the analytical approach introduced here can be used to gain an insight into MammoWave’s detection capability without having to perform either time-consuming full-wave simulation or phantom measurements.

The procedure can be applied at any frequency since it does not resort to low-frequency or quasi-static approximation. However, the average relative error showed a decreasing trend as we increased the frequency; specifically, for 1–2 GHz, we achieved a relative error of 14%, whilst this value decreased to 7% for the largest sub-band of 8–9 GHz. Also, observing microwave images for the individual sub-bands showcases an improvement in target detection and localization with the increase in frequency; this holds true both in the images generated through measurement with the phantom and in the images generated through the analytical simulation approach.

A first limitation is that the simulator is 2D, and that it has only been verified for two-layered cylinders. Also, another main limitation is related to the fact that the simulator does not take into account antennas’ parameters and coupling. Such differences between the simulator and the system, commonly referred to as modeling error, may lead to severe errors in tomographic microwave imaging approaches. Several research groups are working to reduce such modeling errors via appropriate calibration [24,25]. However, it is worthwhile pointing out that the HP-based algorithm is a direct non-quantitative imaging algorithm; this may explain the low relative errors of Figure 3c and Figure 4c.

## Figures and Tables

**Table 1:** Comparison of microwave imaging prototypes in advanced clinical trial stage.

	MammoWave	Dartmouth College	MARIA	Wavelia	SAFE
**Array type**	Synthetic	Synthetic	Hardware	Synthetic	Synthetic
**Geometry**	Cylindrical	Cylindrical	Hemispherical	Cylindrical	Cylindrical
**Antenna**	Horn/Vivaldi	Monopole	Slot	Vivaldi	Vivaldi
**No. of antennas**	2	16	60	21	2
**Frequency (GHz)**	1–9	0.7–1.7	3–10	0.8–4	1–8
**Coupling medium**	None	Liquid	Shell + liquid	Creamy liquid	Shell
**Algorithm**	HP	Tomography	DAS	TR-MUSIC	LSM + FM
**Scan time (min)**	8	2	0.17	15	7
**Largest trial**	4000	400	389	73	115

## Data Availability

The original data presented in the study are available upon request.
